# Study on the application of modified negative pressure wound therapy with instillation in cervical anastomotic leakage after oesophageal cancer surgery

**DOI:** 10.3389/fonc.2025.1644593

**Published:** 2025-12-01

**Authors:** Linrong Zhou, Cui Li, Xuehong Guan, Jing Xie, Wanli Lin, Bomeng Wu, Ying Chen, Jiawei Huang, Lanjuan Gong

**Affiliations:** 1 Department of Thoracic Surgery, Gaozhou People’s Hospital, Maoming, Guangdong, China; 2 Guangdong Oesophageal Cancer Institute Gaozhou Branch, Gaozhou, China; 3 Nursing Department, Gaozhou People’s Hospital, Maoming, Guangdong, China

**Keywords:** negative-pressure wound therapy with instillation, oesophageal cancer, cervical anastomotic leakage, odour management, negative-pressure wound therapy

## Abstract

**Aims:**

This study aims to explore the feasibility and safety of modified negative-pressure wound therapy with instillation (NPWTi) for the treatment of cervical anastomotic leakage (CAL) after oesophageal cancer surgery.

**Methods:**

A retrospective analysis was conducted on 17 patients who developed CAL after oesophageal cancer surgery and received modified NPWTi treatment at our hospital from 2021 to 2024. The primary outcome was the time to healing, defined as the number of days from the initiation of modified NPWTi to complete fistula closure. Secondary outcomes included wound odour improvement and patient comfort.

**Results:**

A total of 17 patient were collected, including 10 men and seven women, with an average age of 73.71 ± 8.01 years. None received neoadjuvant therapy, and no patients had diabetes; preoperative albumin was 38.77 ± 3.58 g/L. The occurrence of CAL was noted at 8.88 ± 3.15 d (95% CI: 7.26 to 10.51 d) post-surgery. The modified treatment was commenced 0–12 d after CAL diagnosis, with the earliest case starting on the day of diagnosis. The mean duration of modified NPWTi was 10.88 ± 6.54 d (95% CI: 7.52 to 14.25 d). For the primary outcome, the time from treatment initiation to complete healing was 15.00 ± 7.26 d (95% CI: 11.27 to 18.73 d), with a minimum of five days. No mediastinal or pleural infections related to NPWTi occurred during the treatment. For the secondary outcomes, a significant improvement in wound odour was observed following the administration of the modified NPWTi (P<0.001), with an improvement rate of 100% (95% CI: 85-100%). During the treatment, the patients reported feeling comfortable and expressed overall satisfaction.

**Conclusion:**

Modified NPWTi demonstrated significant efficacy and convenience in treating CAL after oesophageal cancer surgery, benefiting both patients and healthcare providers with good safety profiles, thus warranting broader clinical application.

## 1 Introduction

Minimally invasive McKeown oesophagectomy is the mainstream surgical approach for oesophageal cancer treatment. Cervical anastomotic leakage (CAL) is a common postoperative complication, with an incidence ranging from 0% to 30% (1). CAL significantly affects patients’ quality of life, including prolonged hospital stay, frequent wound cleaning, unpleasant odour, psychological distress, and potentially life-threatening conditions (2). Although improvements in surgical techniques and perioperative management can reduce the incidence of CAL, this complication has not been entirely eliminated.

In recent years, negative-pressure wound therapy (NPWT) and NPWT with instillation (NPWTi) have emerged as effective methods for accelerating wound healing and are commonly used to treat pressure injuries, diabetic foot ulcers, and chronic osteomyelitis (3, 4). However, reports on the use of NPWTi for CAL after oesophagectomy are sparse, and there is a risk of mediastinal and thoracic infections associated with its use. This paper presents an improved NPWTi method with broad clinical applicability aimed at reducing the risk of fistula infection, promoting wound healing, eliminating odour, reducing frequent dressing changes, and enhancing patients’ quality of life. This study aimed to provide effective clinical management strategies for these common and challenging complications.

## 2 Methods

### 2.1 Design and population

This study is a retrospective analysis of patients who underwent surgery for oesophageal cancer at our hospital between 2021 and 2024. All included patients subsequently developed a cervical anastomotic leak (CAL) and were treated with our modified NPWTi protocol. Patient data, including healing days, treatment frequency, comfort level, wound odour, and patient satisfaction, were collected.

### 2.2 Diagnosis of CAL

A diagnosis of CAL was based on one of the following:

#### 2.2.1 Clinical findings

Local swelling at the cervical incision, elevated skin temperature, and tenderness; upon incision opening, saliva or secretions were observed, accompanied by a foul odour.

#### 2.2.2 Auxiliary examination findings

Gastrointestinal imaging revealed contrast agent overflow; gastroscopy confirmed the fistula.

### 2.3 Outcome measures

The primary and secondary outcomes in this study were as follows:

#### 2.3.1 Primary outcome

##### 2.3.1.1 Time to healing of CAL

This was defined as the number of days from the initial diagnosis of CAL to the commencement of modified NPWTi until the criteria for “Healing of CAL” were met (i.e., after the drainage device was removed, the cervical incision showed no salivary or secretory leakage or foul odour, and gastrointestinal imaging did not reveal any contrast overflow).

#### 2.3.2 Secondary outcomes

##### 2.3.2.1 Wound odour assessment

According to the World Union of Wound Healing Societies consensus document and the *Grocott* wound odour assessment criteria, wound odour is categorised into six levels (5):

- Level 0: Odour detectable upon entering the room.
- Level 1: Odour detectable within the patient’s arm length.
- Level 2: Odour detectable at least one arm’s length away.
- Level 3: Slight odour within arm’s length.
- Level 4: Only the patient could smell it.
- Level 5: No odour.

Wound odour assessment was performed on the day of CAL diagnosis and 1 d after modified NPWTi implementation.

##### 2.3.2.2 Comfort evaluation

The pain assessment visual analogue scale (VAS) was used to evaluate comfort during treatment.

### 2.4 Treatment of CAL

#### 2.4.1 Common treatment

All patients diagnosed with CAL received conventional treatments, including fasting, placement of enteral feeding tubes, enteral and parenteral nutritional support, and anti-infection measures. The cervical incision was fully opened, and dressings were changed intermittently to maintain cleanliness. Contaminants near the incision and anastomosis were thoroughly removed, along with necrotic oesophageal and gastric tissues.

#### 2.4.2 Modified NPWTi

**Figure 1:**
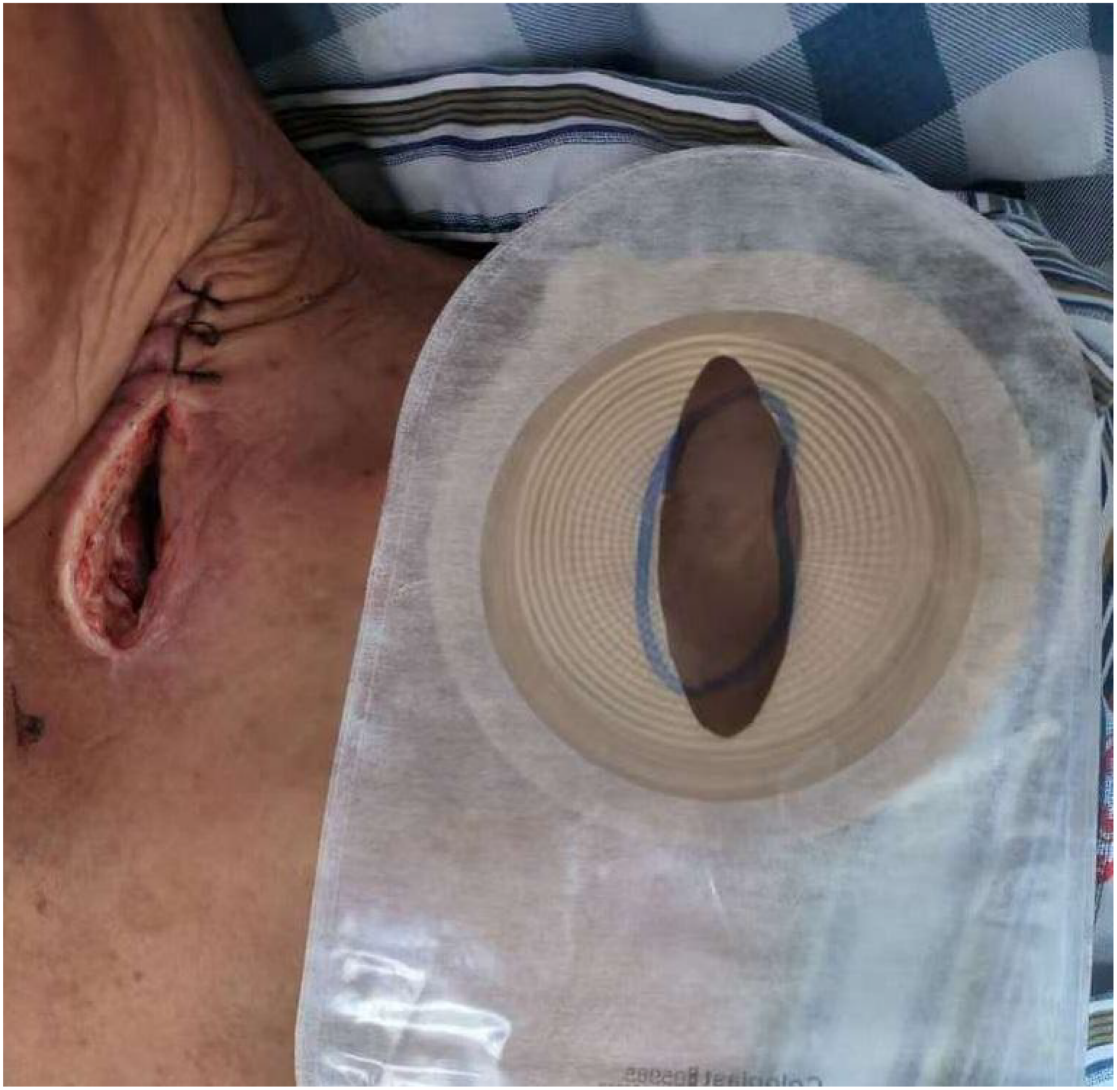
Custom-trimmed stoma bag applied to the incision site. The stoma bag was custom-trimmed to match the size and shape of the incision, ensuring a secure and airtight seal for the subsequent application of negative pressure.

**Figure 2:**
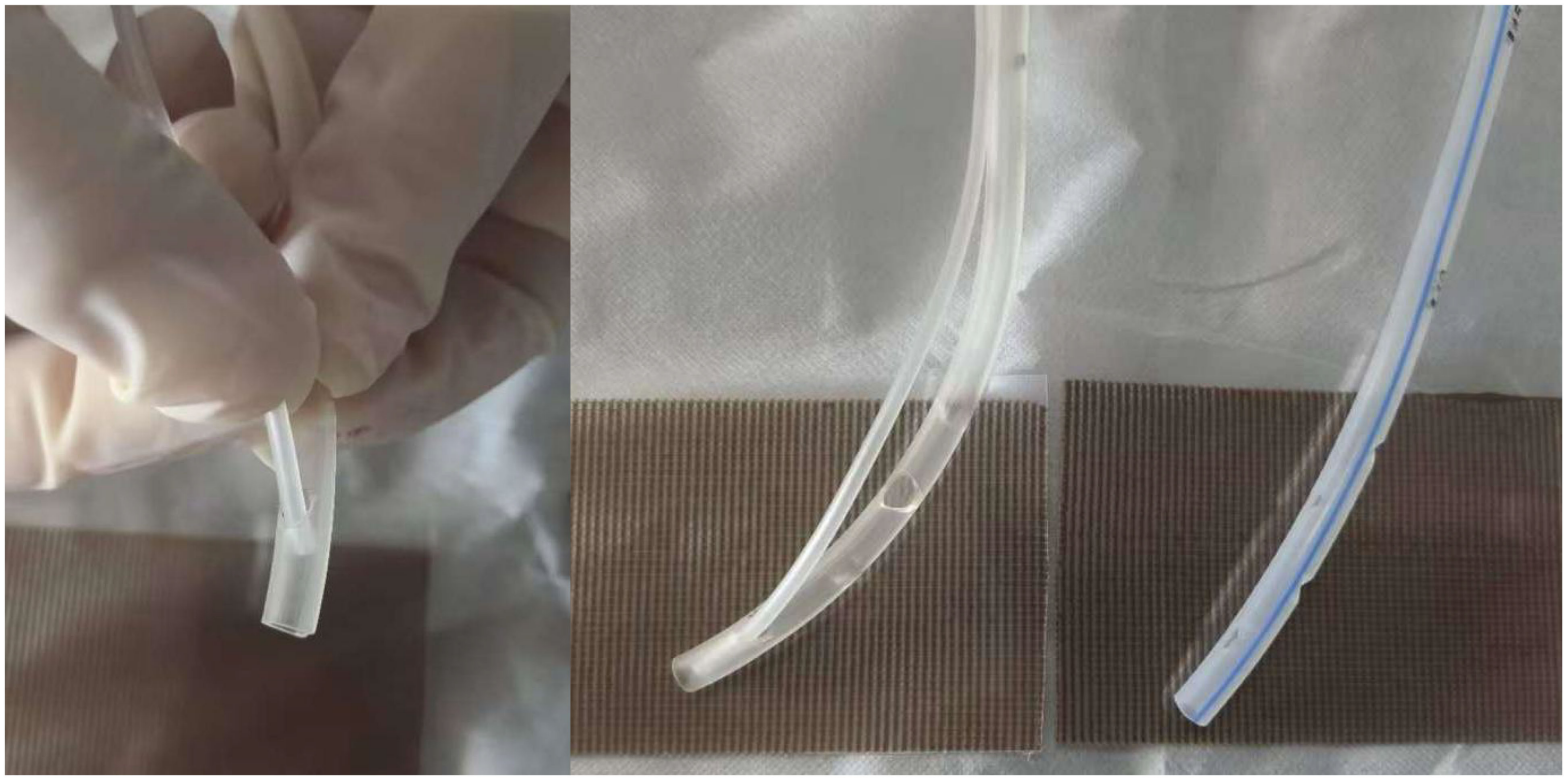
Assembly of the three-tube system. The system is composed of a standard venous infusion catheter and two 14F gastric tubes, which are colour-coded for easy identification. The brown rectangular dressings at the base of the figure are silver sulphate dressings.

**Figure 3:**
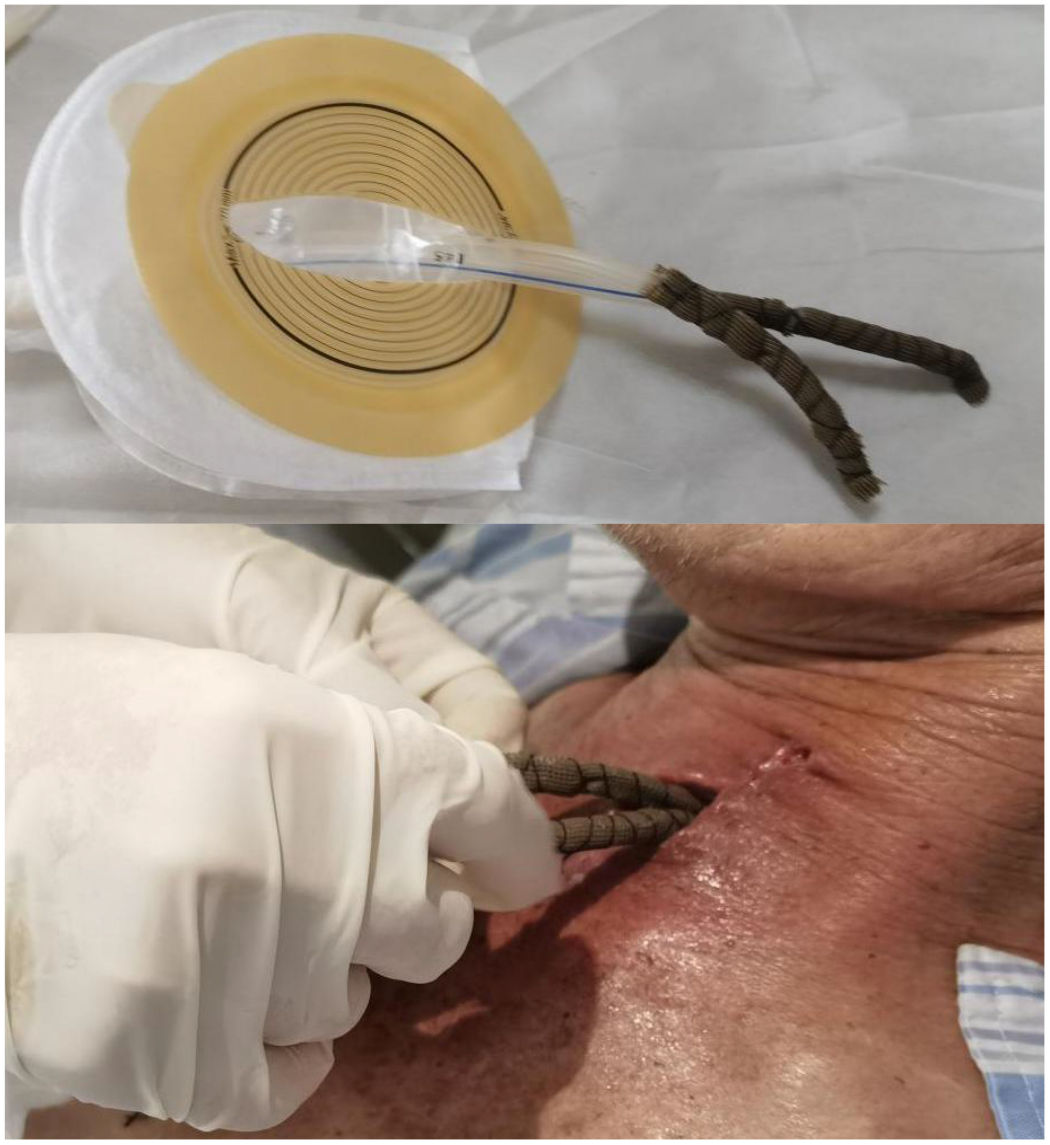
Application of a silver sulphate dressing at the tube-wound interface. A silver sulphate dressing was applied to the exterior of the drainage tube, ensuring its smooth surface was in direct contact with the neck wound.

**Figure 4:**
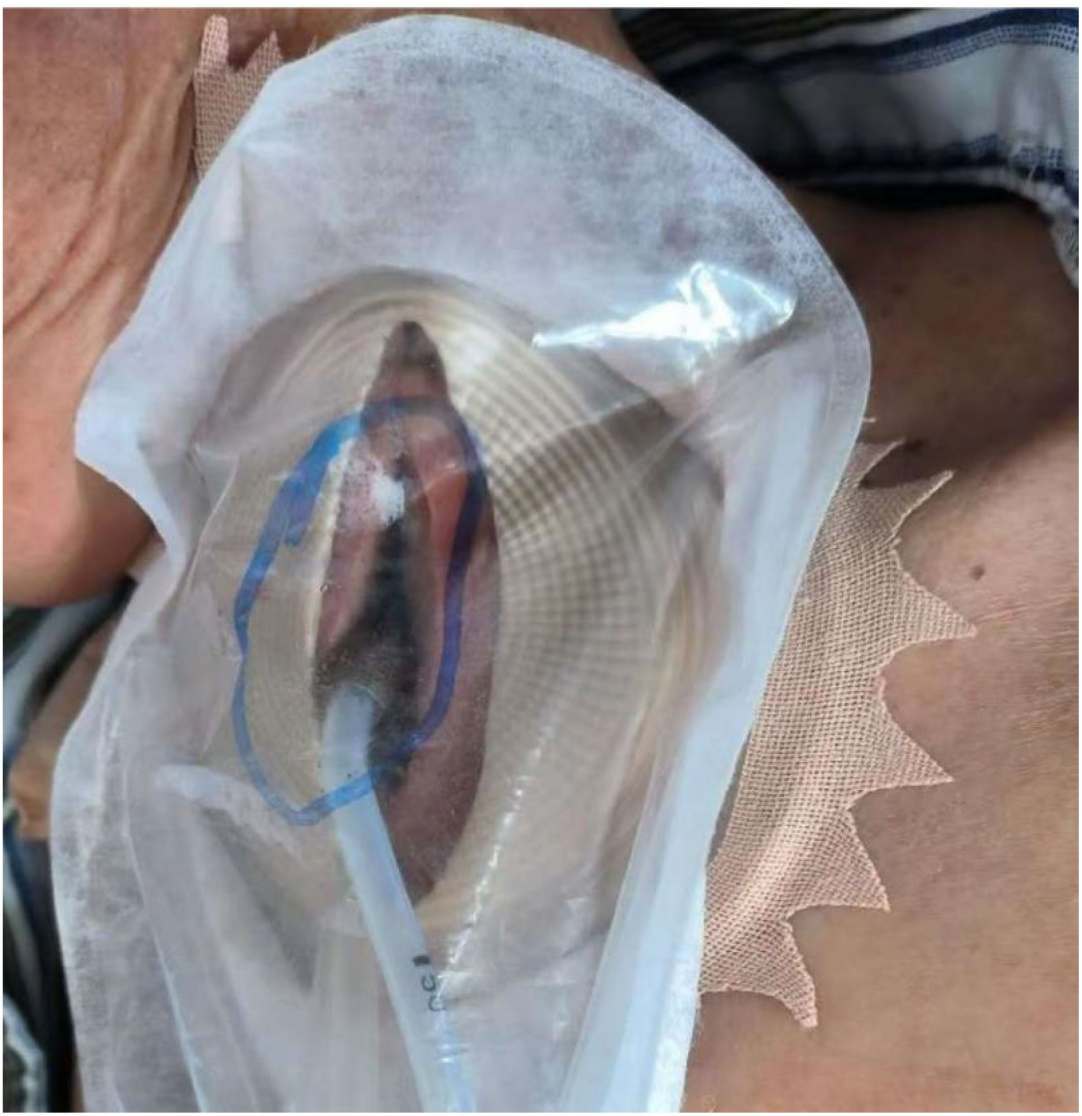
The adhesive dressing is trimmed to create a secure perimeter. The adhesive dressing was trimmed into a serrated edge to conform to the contours of the stoma bag and skin, thus ensuring a complete and hermetic seal across the entire wound area.

**Figure 5:**
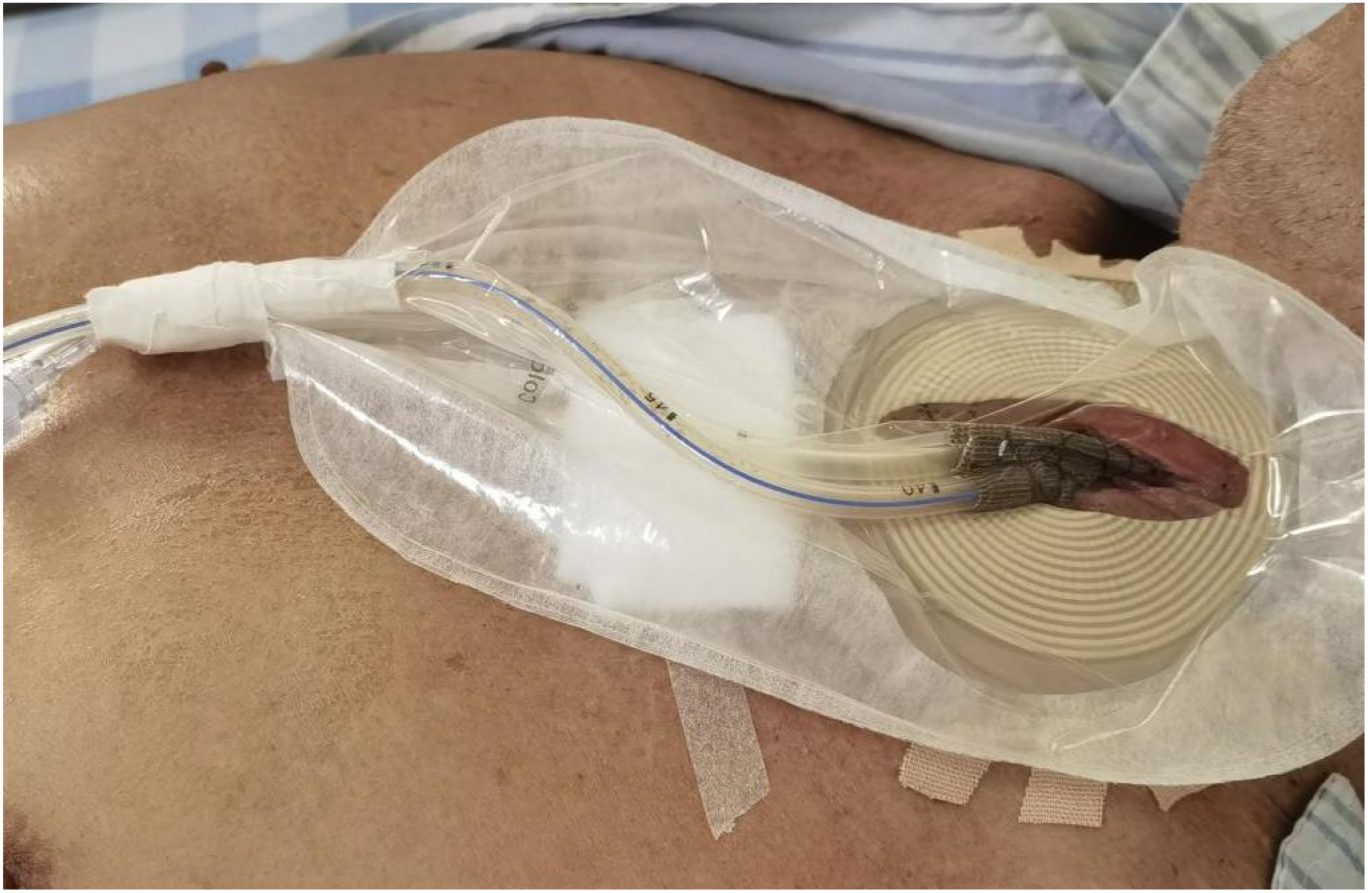
The fully assembled system ready for activation. Following the assurance of a hermetic seal, the initiation of negative pressure suction and medicated infusion via the designated tubes may be undertaken.

1. The stoma bag was trimmed to match the shape and size of the incision (**Figure 1**).

2. A standard venous infusion catheter was inserted into the side hole of a 14F gastric tube (**Figure 2**), with the other end connected to an isotonic sodium chloride solution irrigation line at a drip rate of 20 to 30 drops per minute.

3. The heads of the two 14F drainage tubes were wrapped and secured with a lipid hydrocolloid silver sulphate dressing to ensure smooth contact with the cervical wound (**Figure 3**).

4. The drainage tubes were connected via a three-way device to a drainage bottle, which was linked to the central negative-pressure system. The pressure was maintained at approximately 0.05 MPa and adjusted based on the patient’s subjective tolerance.

5. The adhesive dressing was trimmed to fit the patient’s neck, ensuring a complete seal of the device (**Figure 4**). The continuous infusion and negative-pressure suction were then initiated (**Figure 5**).

### 2.5 Statistical methods

Data were statistically analysed using IBM SPSS Statistics 26.0. Normally distributed continuous variables are presented as mean ± standard deviation; non-normally distributed variables as median and interquartile range; and categorical data as number (percentage). Wound odour scores, being ordinal data, were compared using the Wilcoxon signed-rank test. A *P* value of <0.05 was considered statistically significant.

## 3 Results

A total of 17 patient were collected (**Table 1**), including 10 men and seven women, with an average age of 73.71 ± 8.01 years. None received neoadjuvant therapy, and no patients had diabetes; preoperative albumin was 38.77 ± 3.58 g/L. The occurrence of CAL was noted at 8.88 ± 3.15 d (95% CI: 7.26 to 10.51 d) post-surgery. The modified treatment was commenced 0–12 d after CAL diagnosis, with the earliest case starting on the day of diagnosis. The mean duration of modified NPWTi was 10.88 ± 6.54 d (95% CI: 7.52 to 14.25 d). For the primary outcome, the time from treatment initiation to complete healing was 15.00 ± 7.26 d (95% CI: 11.27 to 18.73 d), with a minimum of five days. No mediastinal or pleural infections related to NPWTi occurred during the treatment. For the secondary outcomes, a significant improvement in wound odour was observed following the administration of the modified NPWTi (P<0.001) (**Table 2**), with an improvement rate of 100% (95% CI: 85-100%). During the treatment, the patients reported feeling comfortable and expressed overall satisfaction.

**Table 1:** Characteristics.

Variable	n=17
Sex (male)	10 (58.8%)
Age (years)	73.71 ± 8.01
BMI (kg/m^2^)	21.64 ± 2.55
Diabetes	0 (0%)
ALB (g/L)	38.77 ± 3.58
Operation duration (min)	345.59 ± 75.24
Neoadjuvant treatment	0 (0%)
Pathological subtype	
Highly differentiated ESCC	0 (0%)
Moderately differentiated ESCC	12 (70.5%)
Poorly differentiated ESCC	4 (23.5%)
Sarcomatoid carcinoma	1 (5.9%)
Pathological stage	
T1	1 (5.9%)
T2	5 (29.4%)
T3	11 (64.7%)
N0	8 (47.1%)
N1	5 (29.4)
N2	3 (17.6%)
N3	1 (5.9%)
VAS score during treatment	
0	2 (11.7%)
1	5 (29.4%)
2	5 (29.4%)
3	5 (29.4%)
Feeding type	
Jejunostomy tube	4 (23.5%)
Nasoduodenal feeding tube	13 (76.5%)
Postoperative time to CAL diagnosis (d)	8.88 ± 3.15
Duration of improved NPWTi (d)	10.88 ± 6.54
Healing time for CAL (d)	15.00 ± 7.26

ALB, albumin; BMI, body mass index; CAL, cervical anastomotic leakage; NPWTi, negative pressure wound therapy with instillation; ESCC, oesophageal squamous cell carcinoma; VAS, visual analogue scale.

**Table 2:** Grocott odour assessment before and after modified NPWTi.

Grocott odour assessment^a^	Before modified NPWTi (n=17)	After modified NPWTi (n=17)
level 0	13 (76.5%)	0 (0%)
level 1	4 (23.5%)	0 (0%)
level 2	0 (0%)	0 (0%)
level 3	0 (0%)	4 (23.5%)
level 4	0 (0%)	6 (35.3%)
level 5	0 (0%)	7 (41.2%)

a Variable *P* value was calculated by Wilcoxon signed-rank test, *P* <0.001.

NPWTi, negative pressure wound therapy with instillation.

## 4 Discussion

### 4.1 CAL after oesophageal cancer operation

Tension, local blood supply, and surgical duration are recognised risk factors for CAL after oesophageal cancer surgery. Additional perioperative factors include neoadjuvant therapy (1, 6), diabetes, body weight (7), and preoperative hypoproteinaemia. Conventional treatment for CAL involves complete opening of the cervical incision, adequate drainage, and daily wound cleaning until healing. Patients typically require fasting, enteral feeding tube placement, nutritional support, and antibiotics. This treatment process not only has a long duration but also increases hospital stay and costs, while frequent dressing changes add to the workload of healthcare staff and create psychological stress for patients.

### 4.2 NPWT and NPWTi

NPWT is a recent therapeutic approach that promotes wound healing. Continuous negative pressure removes wound exudates and sources of infection, prevents the spread of infection, and protects the wound. NPWT promotes neovascularization and the expression of related growth factors (8–10). It is widely used to treat pressure ulcers, leg ulcers, poor postoperative healing, diabetic foot ulcers, and various wounds.

Compared to NPWT, NPWTi enhances wound hydration through a stable irrigation fluid, accelerates wound cleaning, and aids in the dissolution and removal of deep necrotic tissue, thereby reducing the absolute bioburden (11) and improving bacterial clearance. NPWTi significantly reduces healing time for infected wounds compared with that of traditional moist wound healing (29.6 ± 6.5 vs. 13.2 ± 6.8 d, *P* < 0.001) (12).

However, healing in CAL cases is fundamentally characterised by the growth of granulation tissue, forming a sinus with prolonged exposure to digestive fluids and saliva, leading to slow healing (13). A meta-analysis of 11 studies indicated that the average duration of CAL treatment was 34 days (14). In this context, the primary outcome of our study demonstrates that the use of our modified NPWTi was associated with a substantially reduced time to healing of 15.00 ± 7.26 days. This finding is particularly notable when considering the expected healing timeline with standard care. While previous studies have confirmed that NPWT is effective and well-tolerated in CAL treatment (15), the risk of infection dissemination with standard NPWTi remains a concern, which our modifications aimed to mitigate.

### 4.3 Technical refinements to NPWTi for enhanced safety

During surgery, the anatomical positioning of the oesophagus allows the cervical incision to connect with the mediastinum and thoracic cavity. Continuous infusion and irrigation are required to maintain an effective negative pressure and avoid dispersion of the irrigation fluid. However, excessive suction may hinder the effectiveness of NPWTi, necessitating a balance between the infusion and suction. We propose the following modifications:

#### 4.3.1 Three-tube system

A small tube is used to control the infusion speed and is embedded within a larger tube to prevent excessive fluid soaking of the wound. Additionally, dual-channel negative pressure drainage can adequately drain large amounts of exudate and inflammatory necrotic material, thereby preventing the further spread of infection.

#### 4.3.2 Preventing blockage

We believe that the blockage of the suction channel is a significant cause of the spread of infection in NPWTi applications for CAL. Dual-channel suction can prevent system failure in the event of single-channel blockage. We also used a lipid hydrocolloid silver sulphate dressing around the suction catheter tip (**Figure 3**), which filters out larger necrotic debris and utilises silver ions for antibacterial effects, thereby reducing the bacterial bioburden (16–18). Smooth dressings also protect the wound by minimising direct damage from negative-pressure suction.

With our modified NPWTi process, we did not observe any cases of CAL progressing to a mediastinal or pleural infection.

### 4.4 Odor management and patient comfort

As secondary outcomes, wound odour management and patient comfort were areas of significant focus. We utilised Grocott’s wound odour assessment criteria to evaluate patients before and after receiving the modified NPWTi, revealing notable improvements. NPWTi effectively seals the cervical incision, isolating it from the odours of digestive fluids and saliva, a result that was well received by most patients and their families. Furthermore, patients reported comfort during the treatment, with a maximum pain VAS score of 3, and expressed overall satisfaction with the procedure.

### 4.5 Reduce the substantial workload

In our study of 17 patients, 14 required only one session of modified NPWTi, whereas three required two sessions, primarily because of incomplete removal of necrotic oesophageal and gastric tissue post-CAL diagnosis. After initiating NPWTi, the nursing staff regularly cleaned the drainage bottles and monitored changes in the exudate without requiring additional treatment for the cervical incision, significantly reducing the workload.

### 4.6 Limitations and clinical implications

This study has several limitations that should be considered. Firstly, it is a single-centre exploratory investigation with a small sample size and the absence of a concurrent control group, which limits the generalisability and statistical power of its findings. Future validation through prospective, large-scale, multicentre studies is therefore necessary. Secondly, our method’s reliance on the hospital’s central negative pressure system restricts patient mobility. There is a practical trade-off between the wide availability and lower cost of central systems and the mobility and potentially more precise control offered by portable negative pressure devices, which come at a higher cost. This consideration directly impacts resource use and implementation strategy in different clinical settings (15). Furthermore, while the assessment of secondary outcomes like odour improvement was performed using established criteria, the perception of odour remains somewhat subjective. Future research should aim to develop and apply more objective, quantitative assessment tools to minimise potential bias.

Despite these limitations, the modified NPWTi demonstrates promise as a feasible and safe intervention for managing CAL. It is associated with a potentially shorter healing time compared to literature-derived benchmarks for standard care, alongside observed benefits in symptom control and nursing efficiency. These encouraging findings warrant further investigation through robust comparative studies, which are needed to validate the efficacy and cost-effectiveness of this modified approach and to guide its broader clinical implementation.

## 5 Conclusion

The application of modified NPWTi in CAL after oesophageal cancer surgery is effective, simple, safe, and beneficial to both healthcare staff and patients. These outcomes warrant further investigation and indicate its potential for widespread clinical implementation.

## Data Availability

The raw data supporting the conclusions of this article will be made available by the authors, without undue reservation.
